# Identification and Optimisation of Indole-3-Acetic Acid Production of Endophytic Bacteria and Their Effects on Plant Growth

**DOI:** 10.21315/tlsr2023.34.1.12

**Published:** 2023-03-31

**Authors:** Saowapar Khianngam, Pimjai Meetum, Pantipa Na Chiangmai, Somboon Tanasupawat

**Affiliations:** 1 Faculty of Animal Sciences and Agricultural Technology, Silpakorn University, Phetchaburi Information Technology Campus, Phetchaburi 76120, Thailand; 2 Department of Biochemistry and Microbiology, Faculty of Pharmaceutical Sciences, Chulalongkorn University, Bangkok 10330, Thailand

**Keywords:** *Bacillus*, Endophytic Bacteria, *Enterobacter*, Indole-3-Acetic Acid, Plant Root

## Abstract

Indole-3-acetic acid (IAA) is one of the most physiologically active auxins produced by rhizobacteria and is potentially applied for agriculture. Two endophytic bacteria, VR2 and MG9, isolated from the root of *Chrysopogon zizanioides* (L.) collected at Cha-Am, and the leaf of *Bruguiera cylindrica* (L.) Blume collected from a mangrove forest at Ban Laem, Phetchaburi Province, Thailand, were taxonomic characterised based on their phenotypic characteristics and 16S rRNA gene analysis. Strain VR2 was closely related to *Enterobacter hormaechei* CIP 103441^T^ (99.6% similarity), while strain MG9 was closely related to *Bacillus aryabhattai* B8W22^T^ (99.9% similarity). Consequently, they were identified as *Enterobacter hormaechei* and *Bacillus aryabhattai*, respectively. The IAA production of VR2 and MG9 strains are determined and applied to rice seeds for their root and shoot germination. Strains VR2 and MG9 greatly produced a yield of IAA, 246.00 and 195.55 μg/mL in 1,000 μg/mL of L-tryptophan at pH 6 for 48 h. They showed no significant differences in IAA to root and shoot development. However, the bacterial IAA exhibited potential nearby synthetic IAA, which had a significant effect compared to the control. IAA produced from these two strains might preferably trim down the use of synthetic IAA and could contribute to sustainable agriculture.

**Highlights**
- Two endophytic bacteria, strain VR2 and MG9 isolated from the root of *Chrysopogon zizanioides* (L.) (Vetiver Grass) and the leaf of *Bruguiera cylindrica* (L.) Blume were characterised and identified as *Enterobacter hormaechei* and *Bacillus aryabhattai*, respectively.
- Bacterial endophytes, strain VR2 and MG9 produced high yield of IAA, 246.00 and 195.55 μg/mL in 1,000 μg/mL of L-tryptophan, at pH 6 for 48 h.
- The bacterial IAA exhibited potential nearby the synthetic IAA when evaluated for the root and shoot development.

## INTRODUCTION

Endophytic microorganisms were distributed in healthy plants. They exhibited the key roles in producing secondary metabolites, plant growth-promoting, and alleviation disease resistance (Yan *et al.* 2018). *Bacillus* strains are spore-forming bacteria isolated from plant rhizospheres. They showed plant growth and played a role in the growth of corn, soybean, and wheat (Akinrinlola *et al*. 2018). Many *Bacillus* strains produced high Indole-3-acetic acid (IAA) that will be applied for seedling treatment for plant production (Shahid *et al*. 2021). Some *Bacillus* strains isolated from root, leaf and stem of pearl millet exhibited plant growth and produced biological control agents to inhibit *Rhizoctonia solani, Sclerotium rolfsii*, and *Fusarium solani* (Kushwaha *et al*. 2020). Several Gram-negative bacteria including *Enterobacter* and *Pseudomonas* strains should be contained the indole pyruvate decarboxylase (IPDC), which is essential in IAA production. They were reported to produce higher than the same strains without the gene (Koga *et al*. 1994). The *Enterobacteriaceae and Pseudomonas* strains caused cranberry stem gall and were suggested to be caused by the IAA-producing strains (Vasanthakumar & McManus 2004). *Enterobacter asburiae* JAS5 and *E. cloacae* JAS7 showed the ability of plant growth-promoting (PGP) traits such as IAA production, organic acid production, and solubilization of various inorganic phosphates (Abraham & Silambarasan 2015). In addition, *Enterobacter* sp. DMKU-RP206 isolated from rice leaves in Thailand synthesized high-yield IAA (Nutaratat *et al*. 2017). Moreover, IAA from endophytic bacteria is safe. Thus, endophytic bacteria are the source of natural products for plant growth.

Rice is an important crop and a major food source in Thailand. Healthy plants, including organic rice, have been consumed by people, and agricultural practices should be improved to reduce chemical reagents for sustainability and the environment (Anugrah *et al*. 2021). Microbial products are used for plant or rice growth promotion in eco-friendly environments. Several studies reported that endophytic bacteria produced secondary metabolites that could promote plant growth and inhibit phytopathogens. IAA is one of the most physiologically active auxins produced by plants and microorganisms, including bacteria and fungi (Lynch 1985). Its role has been reported for the growth and development of plant parts including cell elongation, cell division, and differentiation in shoots and roots (Taghavi *et al.* 2009; Popko *et al*. 2010; Miransari & Smith 2014). The capacity to produce the phytohormone IAA is widespread among bacteria that inhabit diverse environments such as soils, fresh and marine waters, and plant and animal hosts (Patten *et al*. 2013). Increasing crop yield using the potential of microorganisms as plant growth promotors, bioinoculants, or biofertilizers to decrease the use of chemical fertilizer is still required. The objectives of this study are isolation, identification and IAA production by endophytic bacteria isolated from plant roots and leaves. The optimisation of IAA production and the rice growth promoting effects of IAA are also investigated.

## MATERIALS AND METHODS

### Isolation of Endophytic Bacteria from Plants

The root of *Chrysopogon zizanioides (L.)* (Vetiver Grass) was collected at Cha-Am (12.698901232037029, 99.90373521252211), and the leaf of *Bruguiera cylindrica* (L.) Blume was collected from a mangrove forest at Ban Laem (13.062525, 100.082652), Phetchaburi Province, Thailand. The strains were isolated by using the surface sterilisation technique. Plant parts were cut and put in plastic bags. The collected samples were then kept in an ice box for transportation to the Microbiology Laboratory at the Faculty of Animal Sciences and Agricultural Technology, Silpakorn University. Samples were immediately brought to the laboratory and were used within 24 h. Samples were washed with running water to remove soil particles and subsequently surface-sterilised by immersed in 70% ethanol for 5 min and 2% sodium hypochlorite (Hi-Media, India) for 10 min. Samples were rinsed three times with sterile distilled water to remove chemical agents. Plant parts were excised with the blade, placed on Nutrient Agar (NA) medium (*Difco*, USA), and incubated at 37°C for 2–7 days. Bacterial isolates were picked up, then purified on NA agar and preserved on NA agar slant. One loop of the last rinsing liquid was also streaked on the NA agar to check the surface sterilization efficacy (Cao *et al*. 2004).

### Identification of Strains

Their morphological, cultural, physiological and biochemical characteristics were determined as described by Barrow and Feltham (1993). The 16S rRNA gene sequencing was sequenced with the primers 27F (5′-AGAGTTTGATCCTGGCTCAG-3′) and 1492R (5′-GGTTACCTTGTTACGACTT-3′) by Macrogen Inc. (Seoul, South Korea). The nucleotide sequence of isolates was aligned with selected sequences obtained from GenBank using CLUSTAL_X version 1.83 (Thompson *et al*. 1997). The phylogenetic tree was constructed using the neighbour-joining (Saitou & Nei 1987) algorithms in MEGA4 software (Tamura *et al*. 2007). Confidence values of branches of the phylogenetic trees were determined using bootstrap analyses (Felsenstein 1985) based on 1000 resamplings.

### IAA Production

IAA production was prepared from VR2 and MG9 isolates. For IAA production, each 1% of overnight cultures was inoculated in Nutrient broth (NB) (Difco, USA) supplemented with 100 ug/mL L-tryptophan (Hi-Media, India) and incubated at 30°C for 72 h with 150 rpm. The suspension was centrifuged at 3,000 rpm for 15 min. Then, the supernatant was detected IAA quantity as described by Phetcharat and Duangpaeng (2012) method. NB+100 μg/mL L-tryptophan was used as control.

### Optimisation of IAA Production

IAA production of strain VR9 and MG9 were optimised using different parameters: incubation time, pH, and concentration of L-tryptophan with based on NB supplemented with L-tryptophan and incubated on a rotary shaker (150 rpm) at 30°C. The incubation time at 0 to 72 h, a range of pH 6 to 8, and the concentration of L-tryptophan at 0–1,000 μg/mL were examined for its effect on IAA production. IAA quantification values were estimated using a calibration curve made from the IAA standard (Sigma Aldrich, USA), with 10–100 μg/mL concentration (Phetcharat & Duangpaeng 2012). The experiments were designed by Completely Randomised Design (CRD), and the results were analysed with Analysis of Variance (ANOVA). All experiments were carried out with three replicates. The results were determined and compared the differences between test groups by Duncan’s Multiple Range Test (DMRT) at a 0.05 probability level.

### Determination of IAA using Thin Layer Chromatography (TLC)

After optimal conditions, the bacterial IAA was confirmed using TLC plate (Silica gel 60 F254, Merck, Germany). Supernatant of culture broth was collected. Supernatant was adjusted to pH 2.5 with 1N HCl, mixed with ethyl acetate (1:1) (Sigma Aldrich, USA). The upper layer was collected and evaporated by rotary evaporator at 40°C. The dried extract was dissolved with methanol (Sigma Aldrich, USA) for sample preparation (Bharucha *et al*. 2013). The standard IAA (15 μg/μL) and extracted samples (VR2 and MG9) were spotted on TLC plate. The solvent system was hexane: ethyl acetate: acetic acid (4.5 : 5.0 : 0.5 mL), (Sigma Aldrich, USA) (Modified from Jeyanthi & Ganesh 2013). Spots with R_f_ values identical to authentic IAA and compared with R_f_ of pure IAA as positive control. The R_f_ value was calculated using the equation:


$$\text{Retention factor }({\text{R}}_{\text{f}})=\text{ditance traveled by solute}/\text{distance traveled by solvent}$$

### Plant Growth-Promoting with IAA of Strains

#### Preparation of IAA

IAA from bacterial culture and synthetic IAA were used for plant growth promotion. The supernatant of IAA from bacterial culture was sterilised at 121°C for 15 min. Sterile supernatant and IAA concentration were calculated and used as one factor for testing.

#### Rice seed preparation

The lowland Thai rice variety (RD41) was famous for the farmer and was selected for this experiment. Unhusked rice seeds were soaked with sterile water (Trt1), synthetic IAA (Trt2), 2.5 μM IAA of *Enterobacter hormaechei* VR2 (Trt3), and 2.5 μM IAA of *Bacillus aryabhattai* MG9 (Trt4) for 8 h. Soak seeds were placed to culture on sterile tissue paper box (100 seeds/box), including 4 replications incubated at room temperature for 2 weeks. The data were recorded with various characterisation. Moreover, after incubating for 7 and 14 days, each treatment was sprayed with sterile water, synthetic IAA, 2.5 μM IAA of *E. hormaechei* VR2, and 2.5 μM IAA of *B. aryabhattai* MG9.

#### Characteristic determination

The final percentage of germination (*GP_f_*) and speed (shoot) germination index (*SGI_s_*) were recorded. For the other ten characteristics, which was explained as follows, the characteristics related to germination were recorded within a week after the germination begun. However, the characteristics associated with seedling growth were recorded after 2 weeks of germination. Calculating formulas for various characteristics are as follow:


$$\text{Final percentage of germination }({GP}_{f})=\frac{x_{t}}{X}\times 100$$

When; *x_i_* as germinated seeds number (2 mm of germinated root or shoot was observed) from day 1 of germination until final day; *X* as total seed number for culturing (Abari *et al.* 2011; Belwal *et al*. 2015).


$$\text{Speed }(\text{shoot})\;\text{germination index}\;(SGIs)=\Sigma \frac{s}{\text{n}}$$

Where *s* as total shoot germinated numbers in each day; *n* as total counting days (Abari *et al*. 2011).


$$\text{Time of average germination }(TAG)=\frac{\sum \left(x_{i}\times d_{i}\right)}{\sum x_{i}}$$

Where as germinated seeds number recorded at day *i*, *d* as days for culturing; ∑*x*_i_ as germinated seeds number in total; modified the formula reported by Mavi *et al*. (2010).


$$\text{Germinating of seed rate }(GSR)=\frac{x1+\left(x1+x2\right)+\left(x1+x2+x3\right)+\left(x1+x2+x3+xn\right)}{n\left(x1+x2+x3\right)}$$

Where *x_1_, x_2_, x_3_* as number of germinated seeds in 1st, 2nd, 3rd day of counting; *x_n_* as germinated seeds number in last days for counting; *n* as days number for counting; modified the formula reported by Yousof and El-Saidy (2014).


$$\text{Germinating coefficient }(GC)=\frac{100(x1+x2+\ldots +xn)}{x_{1}d_{1}+x_{2}d_{2}+\ldots +x_{i}d_{i}}$$

Where as the number of germinated seeds at day *i*, *d* as the culturing period in days; modified the formula reported by Yousof and El-Saidy (2014).


$$\text{Vigor index }(VI)=\frac{\left({GP}_{f}\times SL\right)}{100}$$

Where *GP_f_* as germination percentage; *SL* as shoot length; modified the formula reported by Alizadeh *et al*. (2013).

Root scores (*RS*) were evaluated at week two after germination according to the fibrous root’s degrees of density and length. The score of the root was classified 1 to 5 scores, i.e., 1 = very low, 2 = low, 3 = moderate; 4 = quite high, 5 = high. Shoot length (*SL*) and root length (*RL*) were also recorded at two weeks after germination. After that, seedling examples were separated for the shoot and root parts. Each shoot and root part was dried at 60°C for 24 h and weighed when the samples cooled down. Shoot dry weight (*SDW*), and root dry weight (*RDW*) were recorded after weighted, and the ratio of *SDW/RDW* was calculated.

#### Experimental design and statistical analysis

Completely Randomised Designed (CRD) was used in this experiment. One-hundred seeds are put on box and used four replications per treatment. For statistical analysis, all data obtained are analysed by analysis of variance (ANOVA), and the mean differences are compared by Duncan’s Multiple Range Test (DMRT) at a 0.05 probability level.

## RESULTS AND DISCUSSION

### Isolation and Identification of Strains

Strain VR2 was Gram-negative rod-shaped and formed yellow, round, entire, smooth, flat and translucent colonies, while MG9 was Gram-positive rod-shaped and formed white, irregular, lobate, smooth, flat and opaque colonies on NA agar plates. They were facultative anaerobic and grew at pH 5–9 and 40°C. Indole production and hydrolysis of gelatin, starch, Tween 20, Tween 80 and tyrosine were positive. However, they were negative for acid production from D-fructose, D-galactose, D-glucose and lactose, as listed in Table 1.

**Figure 1:**
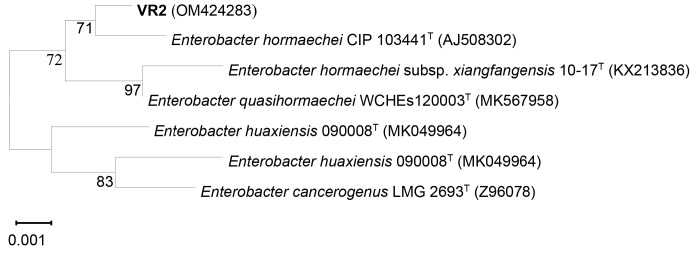
Neighbour-joining tree based on 16S rRNA gene sequences showing the phylogenetic relationships between representative isolates VR2 and Enterobacter species. Based on 1,000 resampling, bootstrap percentages above 50% are shown. Bar, 0.001 substitutions per nucleotide position.

**Figure 2:**
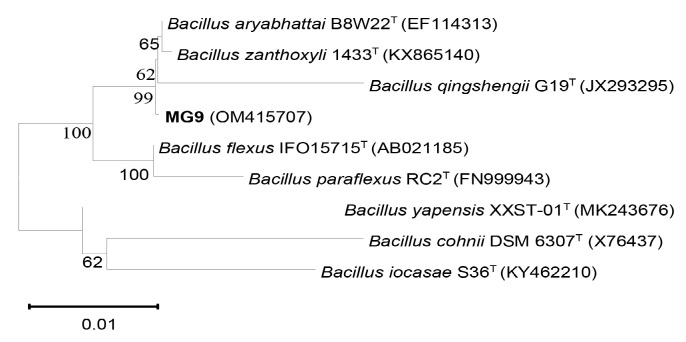
Neighbour-joining tree based on 16S rRNA gene sequences showing the phylogenetic relationships between representative isolates MG9 and Bacillus species. Based on 1,000 resampling, bootstrap percentages above 50% are shown. Bar, 0.01 substitutions per nucleotide position.

Based on 16S rRNA gene sequence and phylogenetic tree analysis, strain VR2 (1,400 bps) was closely related to *Enterobacter hormaechei* CIP 103441^T^ with 99.6% sequences similarity, and strain MG9 (1,422 bps) was closely related to *Bacillus aryabhattai* B8W22^T^ (=*Priestia aryabhattai* B8W22^T^) with 99.9% sequences similarity (Figs. 1 and 2). Therefore, VR2 and MG9 were identified as *Enterobacter hormaechei* and *Bacillus aryabhattai* (=*Priestia aryabhattai*), respectively.

### IAA Production

Various reports presented IAA production from *Enterobacter* and *Bacillus aryabhattai* strains (Bhutani *et al*. 2018). In this study, strain VR2 and MG9 exhibited IAA production, 52.24 and 32.22 μg/mL in 100 μg/mL L-tryptophan, respectively, after incubated on a rotary shaker (150 rpm) at 30°C for 72 h. Our two strains showed a potential source of IAA production. This study is the first report of IAA production from *Enterobacter hormaechei*.

### Optimisation of IAA Production

**Figure 3:**
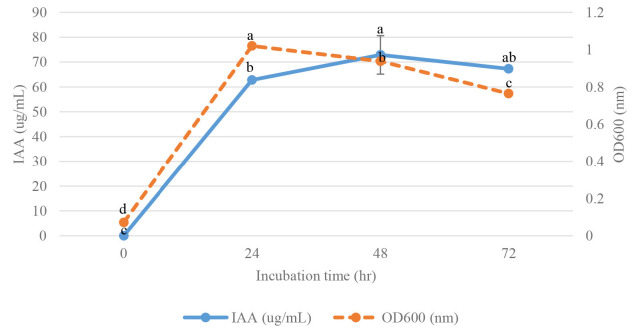
Effect of incubation time for IAA production and growth curve of *Enterobacter hormaechei* VR2. Mean of each parameter with different alphabets indicated significant difference (*p* < 0.05). Data bars represent mean (±SD) for triplicate samples.

**Figure 4:**
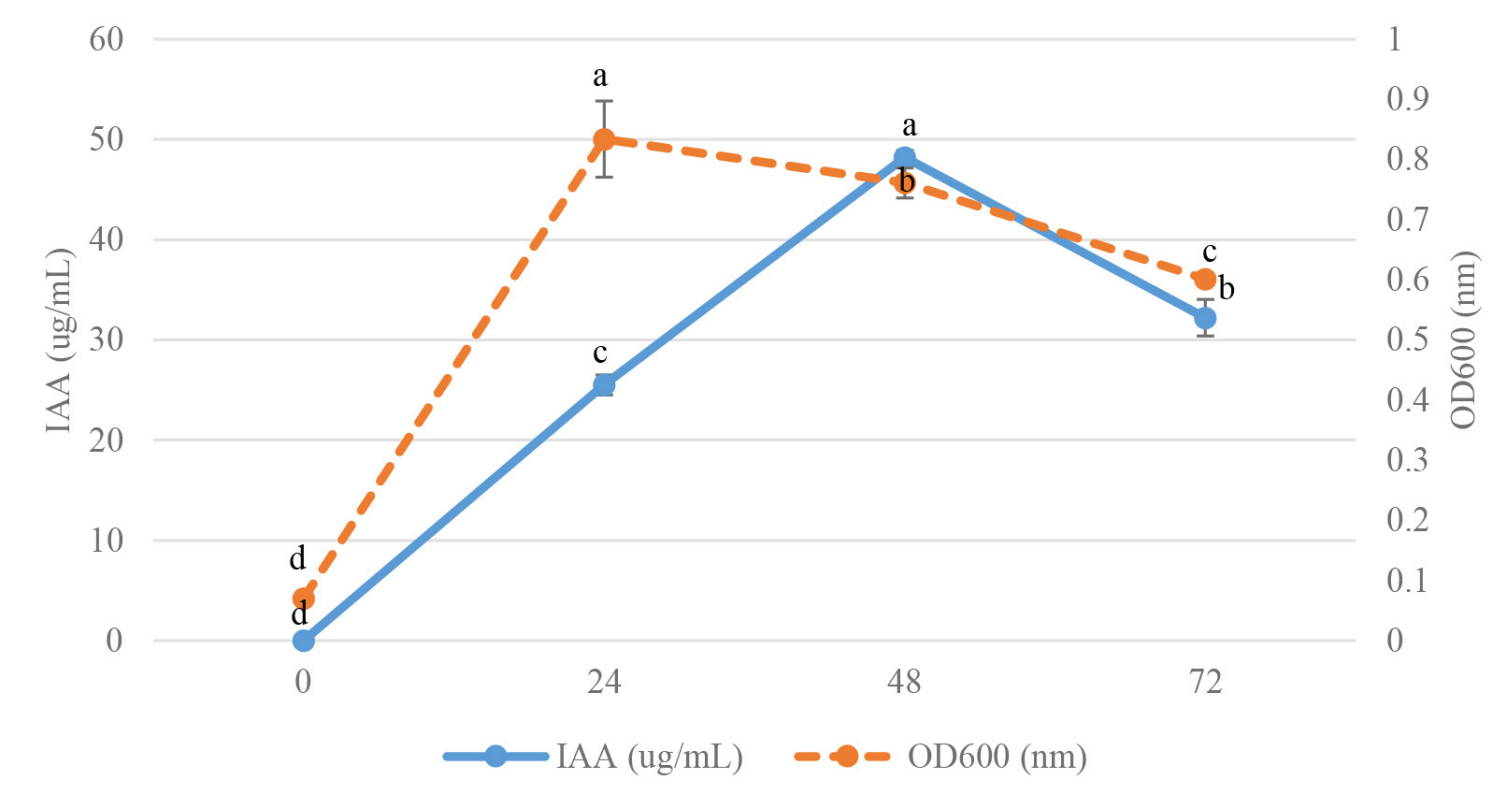
Effect of incubation time for IAA production and growth curve of *Bacillus aryabhattai* MG9. Mean of each parameter with different alphabets indicated significant difference (*p* < 0.05). Data bars represent mean (±SD) for triplicate samples.

**Figure 5:**
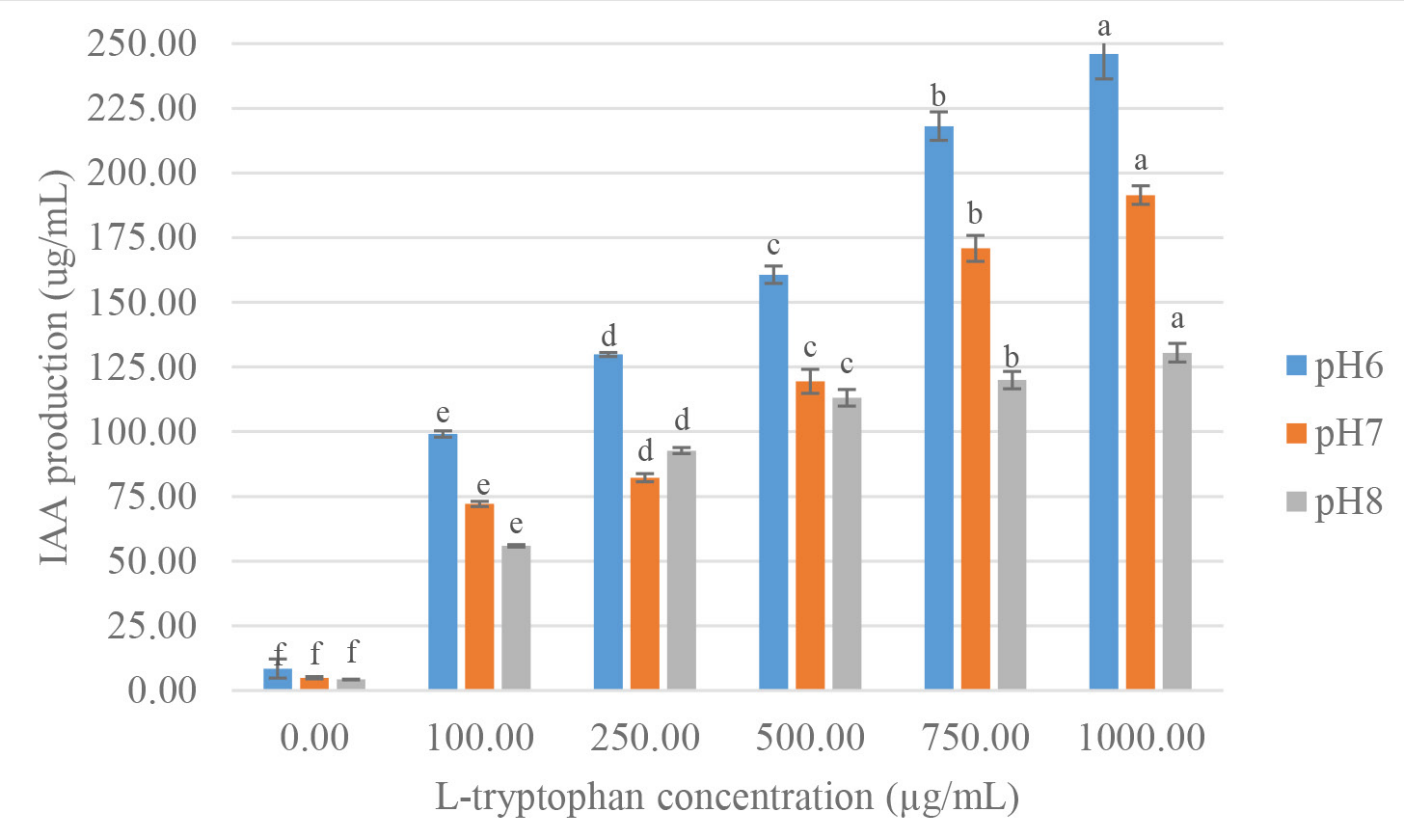
Effect of pH and L-tryptophan concentration for IAA production of *Enterobacter hormaechei* VR2. Mean of each parameter with different alphabets indicated significant difference (*p* < 0.05). Data bars represent mean (±SD) for triplicate samples.

**Figure 6:**
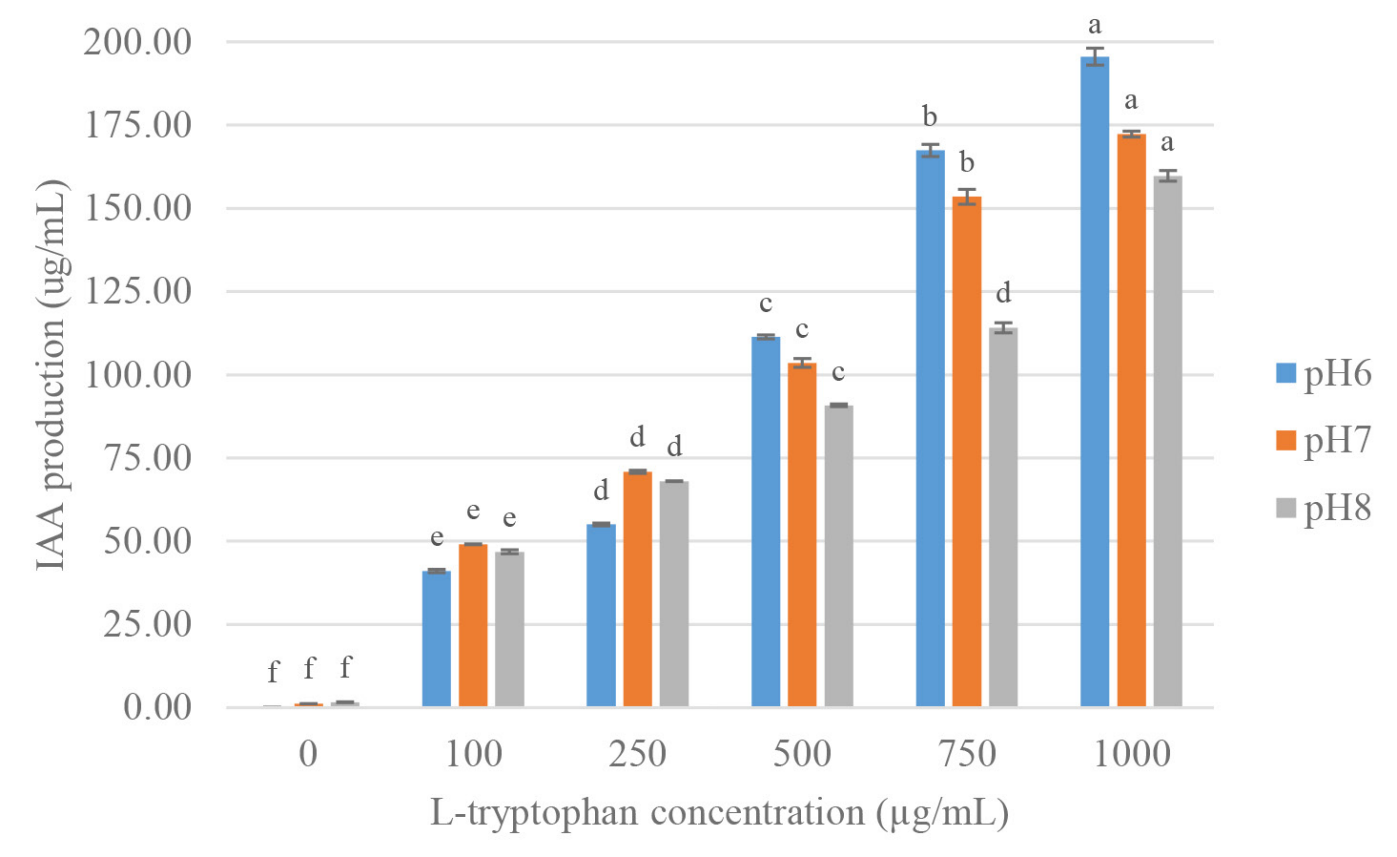
Effect of pH and L-tryptophan concentration for IAA production of *Bacillus aryabhattai* MG9. Mean of each parameter with different alphabets indicated significant difference (*p* < 0.05). Data bars represent mean (±SD) for triplicate samples.

Two strains were cultivated using 1% inoculum in NB medium supplemented with 100 ug/mL L-tryptophan and incubated at 30°C for 0–72 h, at 24 h intervals to study the effect of incubation time on IAA production. The IAA production started at 24 h after incubation and reached the maximum at 48 h, then gradually decreased product with that observed at 72 h. The high yield of IAA of strain VR2 was 72.90 μg/mL, and strain MG9 was 48.21 μg/mL after 48 h incubation which was significant differences (*p* < 0.05) when compared at 0 h, 24 h and 72 h (Figs. 3 and 4). Thus, the time at 48 h was selected for further study. IAA production was strongly synthesised after 24 h, and they grew in the stationary phase. Similarly, the reports of *B. cereus* So3II and *B. subtilis* Mt3b showed that IAA production gradually decreased after 24 h (Wagi & Ahmed 2019; Panigrahi *et al*. 2020). This result showed that IAA was a secondary metabolite which is produced in the stationary phase (Hanh & Mongkolthanaruk 2017). The two strains showed a maximal yield of IAA production at pH 6 in NB medium supplemented with 100 ug/L-tryptophan. They were incubated at 30°C for 48 h, which showed significant differences (*p* < 0.05) when compared to pH 7 and 8 (Figs. 5 and 6). In correlation to other studies, the IAA production of *E. cloacae* OS03 and *B. cereus* So3II was maximum at pH 7 and 37°C, while *B. subtilis* Mt3b showed highly IAA production at 25°C. *Klebsiella* sp. and *Enterobacter* sp. DMKU-RP206 were optimal at pH 6 (Ait Bessai *et al.* 2022) while *B. subtilis* DR2 (KP455653) was at pH 7 (Kumari *et al*. 2018). Thus, the IAA production ability is depended on the isolates.

The results showed that IAA increased when the concentrations of L-tryptophan were increased (Wagi & Ahmed 2019). Then, L-tryptophan was a precursor that influenced IAA production. Thus, the increase of L-tryptophan concentration affected to IAA production of the strains. Strain VR2 produced maximal IAA (246.00 μg/mL) at pH 6 with 1,000 μg/mL of L-tryptophan (Fig. 5) that was increased 4.71-fold compared to the conventional condition. Similarly, strain MG9 showed high IAA production (195.55 μg/mL) at pH 6 with 1,000 μg/mL of L-tryptophan that was increased 6.07-fold (Fig. 6).

### Determination of IAA using Thin Layer Chromatography

**Figure 7:**
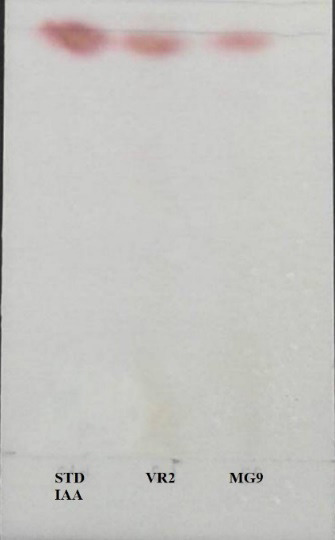
IAA produced by strains VR2 and MG9 as determined by Thin layer chromatography (TLC).

The IAA produced by strains were detected as pink colored spots with R_f_ values 0.86 similar to the IAA standard (Fig. 7) the same as previous reports (Bharucha *et al*. 2013; Mohite 2013).

### Plant Growth-Promoting with IAA of Strains

The germination characteristics were determined in the first week after planting, both on root and shoot. The ratio between GP between shoot and root is related to germination efficacy, including Germination percentage (GP), Mean time of germinating (MTG), Speed germinate index (SGI), Germination rate (GR) and Coefficient of germination (CG). The results were recorded and calculated as shown in Table 2. There were no significant differences in either root or shoot parts, except on the GR of the shoot (Table 2). For GR of the shoot, it showed higher values in treatment 2 (0.98) and treatment 3 (0.97), while treatment 4 showed a lower value (0.96). The GR of the shoot, treatment 1 (or nil water), was found to have the lowest value (0.94). Although other characteristics, excepted in MTG of root and shoot, showed no significant statistical differences among treatments, the values of treatments 2 and 3 seem higher than treatments 1 and 4 (Table 2).

For strain MTG, this characteristic indicates the time used for germination (Maraghni *et al*. 2010). Therefore, the high value means using a long germination period of seed, which is not a good character for seeding. The ratio of GP between shoot and root was about 1 and was not significantly different. This result indicated that the treated seeds with either water or IAA produced by bacterial strains showed the no different effect on seed germination percentage. Moreover, treated seed with either water or IAA produced by bacteria showed the no different effect on shoot germination and root germination (value about 1). The vigor index, which directly measures the strength of shoot growth and the percent germination of the shoot (Alizadeh *et al*. 2013), was not different among treatments in the first week after planting. Although, treatment 2 and 3 showed higher VI values than treatment 1 and 4. For SGI, GR and CG characteristics have the formulas for using both germinated seed numbers and time for germination to calculate. However, some differences in detail of formulas were noted in material and methods (Yousof & El-Saidy 2014). Using the observation of the germinated seed daily to accumulate in the calculation in GR could reduce the deviation of the data in the experiment. Therefore, the likelihood of finding a significant difference between treatments of GR seems higher than in other germination parameters.

Only shoot dry weight exhibited significant differences among six characteristics at one-week-old seedling, including shoot height, root length, root score, shoot and root dry weight, and the dry weight ratio between shoot and root (Table 3). At one week old seedling, for dry shoot weight, the higher dry weight was measured in treatment 4, followed by treatment 3. In addition, it was found that treatments 3 and 4 also were higher values of root dry weight in seedlings, although they were not significantly different from treatment 1 and treatment 2 (Table 3). This observation showed no significant difference among treatments in a dry weight ratio between shoot and root at one-week seedling (Table 2). Besides, this study showed that such characteristics are not influenced by receiving different treatments. Similar values were found in the dry weight ratio between shoot and root. Using 2.5 μM, IAA produced from bacteria is unlikely to affect seedlings’ growth adversely. Because the value obtained from the calculation was not significantly different from spraying with water (in treatment 1) while seeding germination, more than that, the values of the ratio between shoot and root dry weight in all treatments was higher than 1 means the dry weight accumulation in the shooting part is greater than the root part. This expression is an essential aspect of seedling growth. Concerns about the use of hormones at inappropriate concentration are essential issues. The imbalance of IAA will directly affect seedlings’ growth, especially auxin, an important plant hormone for development and its signaling in the vegetative stage (Nakamura *et al*. 2006; Zhang *et al*. 2012; Lavenus *et al*. 2013). Inappropriate use of IAA in plant tissue has been identified as an adverse effect on plant development because many links between phytohormones and several hormones are modulated by the amount and responses of auxin (Woodward & Bartel 2005).

Due to the inverse relationship, cytokinin is a vital plant hormone inhibited by auxin (Eklöf *et al*. 2000; Nordström *et al*. 2004). Moreover, the pleiotropic effects were also observed in overexpression of auxin effects, such as reducing many characteristics, including plant height, leaf number, and either shoot or root biomass (Lu *et al*. 2015). In contrast, the limit of IAA level in roots resulted in the inhibition of root formation (Nordström & Eliasson 1991). Transgenic rice was insensitive to auxin and mutant OsIAA3 protein, and the reduction of crown root formation was reported (Nakamura *et al*. 2006). Nakamura *et al.* (2006) reported that transgenic rice (mutant OsIAA3 protein) was insensitive to auxin; consequently, the root formation of transgenic rice was reduced. At 2.5 μM IAA, this concentration is derived from studying the optimal concentration of IAA to promote the growth of seedlings in previous studies. Although, the IAA hormone used in previous studies is derived from the production of different strains of bacteria. Appropriate balancing of IAA, both on the amount and transporting, between shoot apex and root part to prevent IAA accumulation in plant tissue (Nordström & Eliasson 1991; Woodword & Bartel 2005; Mockaitis & Estelle 2008).

At two weeks old seedling, all six characteristics of shoot and root were increased, including shoot height, root length, root score, shoot, and root dry weight, and the dry weight ratio between shoot and root (Table 4). The higher values were observed in treatment 3 more than in other treatments in all characteristics, except the dry weight ratio between shoot and root. In treatment 3, both the root and shoot parts were positively affected, compared with treatment 4 and treatment 1 (nil water). Moreover, the shoot and dry root weight ratio was higher in treatment 3 than in treatment 2 and control. However, all values were more than 1.0. This result means the application of IAA produced from bacteria at 2.5 μM showed the accumulating of dry weight in shoot part more than root part, which more than that applied by synthetic IAA in treatment 2 and nil water in treatment 1. Auxin is a plant hormone that has been reported in many roles for growth and development in plant parts such as shoots and roots (Popko *et al*. 2010; Miransari & Smith 2014). In treatment 4, although the ratio between shoot and dry root weight had a value of more than 1, the scale of increasing values in both shoot and root characteristics was lower than received in treatment 3. The effect of IAA application was observed at two weeks old seedling, showing significant differences in all characteristics. Only shoot dry weight was significantly different in seedlings at one week old. However, the effect of IAA application was not observed at the seed germination stage (only found significantly different in GR of the shoot).

The delayed response on the root part of the seedling after applying IAA produced by exogenous bacteria can explain by this evidence. Lwin *et al*. (2012) found that the effect of bacterial IAA production affected root (root weight, root length, and the number of adventitious roots) and shoot (shoot weight and shoot height) characteristics on the 20th day after sowing but was not observed at seed germination stage. However, auxin is present in the root part at the seed radicle tip both during and after germination. Auxin was reported to be not a necessary hormone for seed germination because seeds can germinate during the inhibition of auxin synthesis (Miransari & Smith 2014). However, the indirect effect of IAA may occur in its interaction with gibberellins and ethylene, promoting seed germination and development (Chiwocha *et al*. 2005). For this reason, it may result in no significant effect of exogenous IAA application in seeds measured from germination parameters. In contrast to the seedling stage, IAA is significant and necessary; it has many roles, such as cell cycling, growth and development, and establishment of various plant tissue and especially in the role of signaling pathways (Liu *et al*. 2007; Hentrich *et al*. 2013; Miransari & Smith 2014).

However, at 2.5 μM IAA did not significantly affected to plants but the data are interesting to useful with plant that replace on synthetic IAA. This result was trendy to save cost from chemical IAA and safe to consumer and environment.

## CONCLUSION

Strain VR2 and MG9 were isolated from parts of plant tissue and identified as *Enterobacter hormaechei* and *Bacillus aryabhattai*, respectively. These endophytic bacteria had a beneficial effect on plants through their IAA production. Moreover, they showed a high yield of IAA when L-tryptophan was increased under optimal conditions. However, at 2.5 μM IAA from the two strains showed no significant differences in root and shoot development but was not harmful to the plant. The potential IAA production strains could be applied to enhance agricultural production. At least, bacterial IAA can be used to replace chemically synthetic IAA, which was the choice of sustainable agriculture. However, we used only at 2.5 μM IAA in this study, therefore, the optimal IAA concentration for other plants is interesting for future work to increase efficiency with plant growth promotion.

## Figures and Tables

**Table 1:** Phenotypic and biochemical characteristics of isolates VR2 and MG9.

Characteristics	VR2	MG9
Cell form	Rods	Rods
Gram-stain	−	+
Colony colour	Yellow	White
Methyl red	+	−
Voges-Proskauer	+	−
Simmons’ citrate	+	−
L-arginine hydrolysis	+	−
Growth at 10°C	+	−
Growth at 45°C	−	+
Acid production from:		
L-Arabinose	+	−
D-Maltose	+	−
D-Mannitol	+	−
Sucrose	+	−
D-Xylose	+	−

*Note*: + = positive reaction; − = negative reaction.

**Table 2:** Comparison of root and shoot germination parameters of lowland rice seed soaked with exogenous IAA of isolates after one week of sowing.

Treatment	GP^1^_root (%)	MTG^2^_root	SGI^3^_root	GR^4^_root	CG^5^_root	GP^1^_shoot (%)	MTG^2^_shoot	SGI^3^_shoot	GR^4^_shoot	CG^5^_shoot	Ratio GP^1^ (shoot/root)	VI^6^
Trt 1	83.0 ± 4.2	2.3 ± 0.3	38.6 ± 3.5	0.96 ± 0.04	44.7 ± 4.7	79.2 ± 9.1	2.4 ± 0.1	35.0 ± 5.8	0.94 ± 0.03 ^b^	42.1 ± 2.8	1.06 ± 0.11	4.8 ± 0.7
Trt 2	89.5 ± 2.6	2.1 ± 0.0	43.5 ± 0.9	0.98 ± 0.01	47.8 ± 1.1	89.5 ± 2.6	2.1 ± 0.0	43.0 ± 1.0	0.98 ± 0.01 ^a^	46.8 ± 0.6	1.00 ± 0.00	5.3 ± 0.3
Trt 3	85.5 ± 9.1	2.1 ± 0.3	40.3 ± 6.4	0.97 ± 0.04	46.5 ± 4.4	81.8 ± 15.9	2.2 ± 0.1	38.7 ± 8.6	0.97 ± 0.01 ^a^	45.8 ± 2.1	1.06 ± 0.12	5.1 ± 1.1
Trt 4	77.5 ± 12.0	2.1 ± 0.1	37.2 ± 7.0	0.97 ± 0.02	46.8 ± 2.7	73.2 ± 16.1	2.2 ± 0.1	34.4 ± 8.7	0.96 ± 0.02 ^ab^	45.5 ± 2.8	1.07 ± 0.08	4.4 ± 1.0
Mean	83.9	2.2	39.9	0.97	46.5	80.9	2.2	37.8	0.96	45.0	1.05	4.9
P-value (F-test)	0.241^ns^	0.665^ns^	0.381^ns^	0.595^ns^	0.659^ns^	0.35^ns^	0.0615^ns^	0.300^ns^	0.042*	0.0584^ns^	0.684^ns^	0.519^ns^
CV(%)	9.47	9.13	12.72	3.14	7.60	15.16	4.92	17.88	2.01	5.03	8.53	16.98

*Notes:* Trt 1 = nil water (control treatment), Trt 2 = synthetic IAA, Trt 3 = exogenous IAA produced by VR2 at 2.5 μM, Trt 4 = exogenous IAA produced by MG9 at 2.5 μM.

1 GP = germinated percentage;

2 MTG = mean time of germinate;

3 SGI = speed germinate index;

4 GR = germination rate;

5 CG = coefficient of germinate;

6 VI = vigor index.

ns = means non significant difference at 0.05 level of proability.

* = means significant different at 0.05 level of proability.

Different upper case letters **(**a, b**)** in the same column means significant difference at 0.05 level of probability.

**Table 3:** Comparison of shoot and root parameters of lowland rice seedling soaked and sprayed with exogenous IAA of isolates at one week seedling.

Treatment	Shoot height (%)	Root length (cm)	Root scoreǂ	Shoot dry weight	Root dry weight	Ratio of dry weight (Shoot/root)
Trt 1	6.01 ± 0.26	6.79 ± 0.47	0.43 ± 0.04	0.0027 ± 0.0001^c^	0.0026 ± 0.0001	1.06 ± 0.13
Trt 2	5.93 ± 0.24	6.61 ± 0.42	0.44 ± 0.04	0.0027 ± 0.0001^c^	0.0025 ± 0.0001	1.13 ± 0.04
Trt 3	6.18 ± 0.15	7.05 ± 0.46	0.43 ± 0.03	0.0030 ± 0.0001^b^	0.0028 ± 0.0002	1.10 ± 0.03
Trt 4	6.06 ± 0.09	5.84 ± 0.84	0.42 ± 0.04	0.0032 ± 0.0001^a^	0.0029 ± 0.0004	1.14 ± 0.16
Mean	6.05	6.57	0.43	0.0029	0.0027	1.109
*p*-value (F-test)	0.36^ns^	0.06^ns^	0.93^ns^	0.0023**	0.0826^ns^	0.469^ns^
CV(%)	3.30	8.78	8.57	5.01	8.28	7.57

*Notes*: Description for the treatments is shown in Table 2.

ǂ = Logarithm was taken for root score parameter.

ns = means non significant difference at 0.05 level of proability.

** = means significant different at 0.01 level of proability.

Different upper case letters (a, b, c) in the same column means significant difference at 0.05 level of probability.

**Table 4:** Comparison of shoot and root parameters of lowland rice seedling soaked and sprayed with exogenous IAA of isolates at two weeks seedling.

Treatment	Shoot height (%)	Root length (cm)	Root scoreǂ	Shoot dry weight	Root dry weight	Ratio of dry weight (Shoot/root)
Trt 1	6.43 ± 0.87^c^	6.72 ± 0.26^ab^	0.434 ± 0.053^ab^	0.0038 ± 0.0008^b^	0.0034 ± 0.0004^ab^	1.130 ± 0.099^c^
Trt 2	7.67 ± 0.50^b^	6.56 ± 0.69^b^	0.438 ± 0.050^ab^	0.0046 ± 0.0004^b^	0.0036 ± 0.0006^a^	1.317 ± 0.112^bc^
Trt 3	9.90 ± 0.68^a^	7.57 ± 0.17^a^	0.486 ± 0.031^a^	0.0058 ± 0.0003^a^	0.0040 ± 0.0001^a^	1.484 ± 0.091^ab^
Trt 4	7.01 ± 0.86^bc^	5.19 ± 0.80^c^	0365 ± 0.040^b^	0.0042 ± 0.0007^b^	0.0027 ± 0.0005^b^	1.587 ± 0.186^a^
Mean	7.84	6.50	0.431	0.0046	0.0035	1.382
*p*-value (F-test)	0.000244**	0.000873**	0.0168*	0.0022**	0.0192*	0.0046**
CV (%)	9.31	8.75	10.11	11.68	12.56	8.90

*Notes*: Description for the treatments is shown in Table 2.

ǂ = Logarithm was taken for root score parameter.

*,** means significant different at 0.05 and 0.01 levels of proability, respectively.

Different uppercase letters (a, b, c) in the same column means significant difference at 0.05 level of probability.
